# Capsaicin and Proton Differently Modulate Activation Kinetics of Mouse Transient Receptor Potential Vanilloid-1 Channel Induced by Depolarization

**DOI:** 10.3389/fphar.2021.672157

**Published:** 2021-05-20

**Authors:** Kaori Takahashi, Kentaro Araki, Hideo Miyamoto, Rikimaru Shirakawa, Takashi Yoshida, Minoru Wakamori

**Affiliations:** ^1^ Division of Molecular Pharmacology and Cell Biophysics, Department of Disease Management Dentistry, Graduate School of Dentistry, Tohoku University, Sendai, Japan; ^2^ Division of Pharmacology, Faculty of Pharmaceutical Science, Teikyo Heisei University, Tokyo, Japan

**Keywords:** voltage-dependency, patch-clamp techniques, capsaicin, protons, kinetics, TRPV1 channel

## Abstract

The transient receptor potential vanilloid type 1 (TRPV1) channel is a non-selective cation channel expressed with transient receptor potential ankyrin type 1 (TRPA1) in small and medial size neurons of the dorsal root ganglions and trigeminal ganglions. TRPV1 is activated by capsaicin, thermal stimuli higher than 43°C, mechanical stress, and protons (H^+^). Although the TRPV1 channel does not have positively charged residues at regular intervals on its transmembrane segments, alterations in membrane potential also affect the state of TRPV1 channel. In the presence of capsaicin, voltage-dependent probability of opening of the TRPV1 channel and its kinetics have been examined, but the characteristics in the low pH remain unclear. To understand the voltage-dependency of the TRPV1 channel activation, we recorded capsaicin- and proton-induced mouse TRPV1 channel currents in a heterologous expression system. Outward current evoked by depolarizing square pulses in the presence of capsaicin or protons was fitted to a two-exponential function with a time-independent component. The voltage-dependent changes in amplitude of the three components displayed shallow curves and the changes in their ratio to the total current display similar tendencies in the presence of capsaicin and under the low pH. However, the fast and slow time constants in the presence of capsaicin were respectively 5- and 8-fold lower than those obtained under low pH conditions. These results suggest that the TRPV1 channel slowly drives the feed-forward cycle of pain sensation, and capsaicin and protons differently modulate the voltage-dependent TRPV1 channel gating.

## Introduction

Members of the transient receptor potential (TRP) channel superfamily play pivotal roles in sensory physiology. Some TRP channels detect changes in surrounding environments and transduce the signals, while others augment intracellular signals. Currently, 28 human TRP channels have been identified. Based on amino acid sequence comparisons, these channels are subdivided into 6 subfamilies: TRP canonical, TRP vanilloid (TRPV), TRP melastatin, TRP polycystin, TRP mucolipin, and TRP ankyrin (TRPA) (Clapham et al., 2005; Owsianik et al., 2006).

The TRPV type 1 (TRPV1) channel is a non-selective cation channel expressed in small and medial size neurons of the dorsal root ganglions and trigeminal ganglions (Caterina et al., 1997, 1999; Akopian et al., 2007; Bourinet et al., 2014), where they receive noxious information. TRPV1 is a receptor that is activated polymodally not only by capsaicin but also by thermal stimuli higher than 43°C, mechanical stress, and protons (H^+^) (Caterina et al., 1997; Tominaga et al., 1998). Since the TRPV1 channel contains six transmembrane domains (S1-S6) like the voltage-gated Na^+^, K^+^, and Ca^2+^ channels, the TRPV1 channel opens in a voltage-dependent manner (Gunthorpe et al., 2000). The voltage-dependence of TRPV1 channel activation in the presence and absence of capsaicin has been examined previously. Capsaicin shifts the voltage-dependent activation curve in the hyperpolarizing direction with the constantly activated component (Nilius et al., 2005; Matta and Ahern, 2007). Activation kinetics in the presence of capsaicin was examined using a two-microelectrode system in *Xenopu*s oocytes (Ahern and Premkumar, 2002) and using a patch-clamp system in a mammalian expression system (Gunthorpe et al., 2000), but has not been examined in detail in the presence of protons, a native agonist. In the present study, we recorded TRPV1-induced outward currents in human embryonic kidney (HEK) 293 cells that heterologously express the mouse TRPV1 channel and compared the voltage-dependent activation kinetics in the presence of capsaicin and under the low pH. In the presence of capsaicin or protons, an outward TRPV1 channel current develops in a time-dependent manner in response to a square pulse, but opening of the TRPV1 channel at low pH is slower than in the presence of capsaicin.

## Materials and Methods

### Recombinant Expression in HEK293 Cells

HEK293 cells (RIKEN BioResource Research Center, Tsukuba, Ibaraki, Japan, RCB Cat# RCB1637, RRID: CVCL_0045) were cultured in Dulbecco’s modified Eagle’s medium (Sigma-Aldrich, St. Louis, MO, United States) containing 10% fetal bovine serum (Thermo Fisher Scientific, Waltham, MA, United States), 30 U mL^−1^ penicillin (Meiji Seika Pharma, Tokyo, Japan), and 30 μg mL^−1^ streptomycin (Meiji Seika Pharma). HEK293 cells (with a density of 100,000/well) were co-transfected with the vector 1.0 μg pCI-neo or 1.0 μg pCI-neo-mouse TRPV1 and 0.1 μg pEGFP-F (Clontech, Mountain View, CA, United States) as a marker. Transfection was carried out using SuperFect Transfection Reagent (QIAGEN, Hilden, Germany) according to the manufacturer’s instructions. HEK293 cells were trypsinized and plated onto glass coverslips 18 h after transfection. Then cells were subjected to measurements 12–42 h after plating on the coverslips.

### Electrophysiology

Currents from HEK293 cells on the coverslips were recorded at room temperature (22–25°C) using the whole-cell mode of the patch-clamp technique (Hamill et al., 1981) with an EPC-10 amplifier (HEKA Elektronik, Bellmore, NY, United States). Patch pipettes were made from borosilicate glass capillaries (1.5-mm outer diameter, 0.87-mm inner diameter; Hilgenberg, Malsfeld, Germany) using a model P-97 Flaming-Brown micropipette puller (Sutter Instrument, Novato, CA, United States). Pipette resistance ranged from 2 to 3 MΩ when pipettes were filled with the pipette solution described below. The series resistance was electronically compensated to >60%, but the remaining series resistance artifacts were included. Currents were sampled at 50 kHz after low pass filtering at 9.8 kHz (6-pole Bessel pre-filter). Data were collected and analyzed using PATCHMASTER (HEKA Elektronik). The pipette solution contained: 140 mM CsCl, 10 mM EGTA, and 10 mM HEPES, and the pH was adjusted to 7.2 with CsOH. The external solution, ‘0Ca^2+^-1EGTA Tyrode solution’, contained: 145 mM NaCl, 5 mM KCl, 1 mM MgCl_2_, 1 mM EGTA, 10 mM glucose, and 10 mM HEPES, and the pH was adjusted to 7.4 with NaOH. The external solutions with pH 6.0 and 5.5 were prepared by the replacement of equimolar HEPES with MES. The osmolarity of the pipette solution and external solutions was approximately 300 mOsm. The pipette solution and the external solutions were prepared every day. Rapid exchange of the external solutions was conducted using a modified “Y-tube” method (Wakamori et al., 1998b). The exchange of external solution surrounding the HEK293 cells was completed within 1 s. Each day, capsaicin (Wako, Osaka, Japan) was first dissolved in dimethyl sulfoxide at a concentration of 10 mM. Thereafter, this stock solution was diluted with external solution to obtain the appropriate concentration of capsaicin just before use. Dimethyl sulfoxide itself had no effect on the TRPV1 channel at concentrations lower than 0.1%. To prevent contamination with capsaicin, we washed the “Y-tube” system with ethyl alcohol and distilled water and changed the recording chamber (culture dish, Falcon #351008).

### Statistical Analysis

All data are expressed as the means ± S.E. The statistical analysis was made using Student’s *t*-test for two groups or two-way repeated-measures analysis of variance (ANOVA) followed by Tukey’s multiple comparisons tests for three groups with GraphPad Prism 8.0 (GraphPad Software). Significant differences were considered at *p < 0.05.

## Results

We recorded the TRPV1 channel current in GFP-positive HEK293 cells under voltage-clamp conditions using the whole-cell mode of the patch-clamp technique. K^+^ was replaced with Cs^+^ in the pipette solution to block the voltage-gated K^+^ channel. Since Ca^2+^-dependent desensitization occurs for the TRPV1 channel (Caterina et al., 1997; Tominaga et al., 1998), we used ‘0Ca^2+^-1EGTA Tyrode solution’ and recorded the currents for a longer period of time.

### Current-Voltage Relationship of Transient Receptor Potential Vanilloid-1 Channel in the Presence of Capsaicin

**FIGURE 1:**
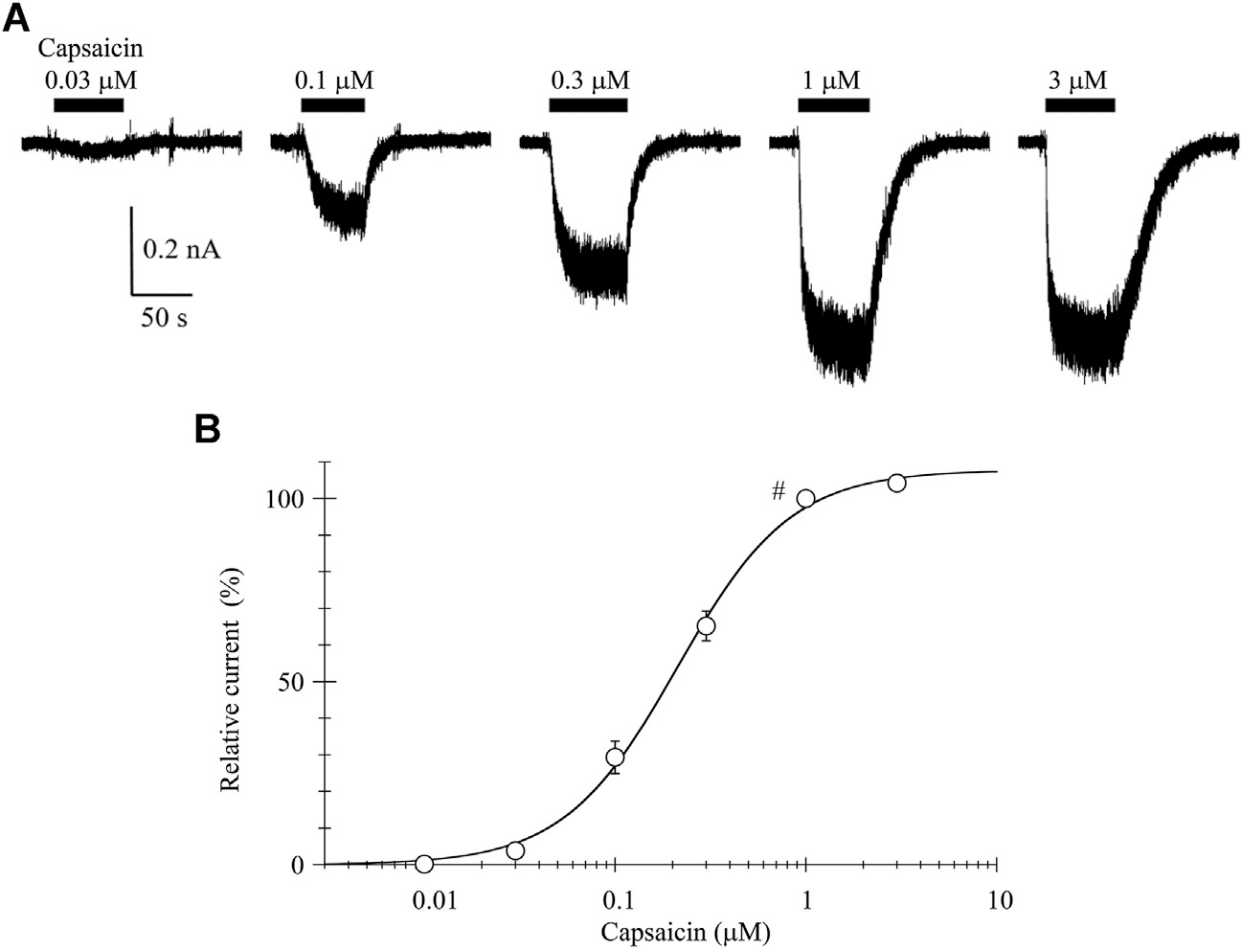
Capsaicin-induced inward currents. **(A)** Representative whole-cell currents induced by capsaicin at a V_H_ of −60 mV. Bars above the traces indicate the application period of capsaicin at different concentrations. **(B)** The concentration-response relationship of capsaicin. Peak current amplitudes were normalized to that induced by 1-μM capsaicin (#). Results are shown as the mean ± S.E (*n* = 6). Curves was drawn by Eq. 1. *I*_max_, EC_50_, and n was 108%, 0.21 μM, and 1.46, respectively.

The 0.03-μM capsaicin induced a small inward current at a holding potential (V_H_) of −60 mV. The 0.1-, 0.3-, and 1-μM capsaicin induced larger inward currents in a concentration-dependent manner with increasing fluctuation (Figure 1A). The 3-μM capsaicin induced the similar current to that induced by 1-μM capsaicin. The current did not decline during a continuous application of capsaicin, but the current level slowly returned to the baseline after washout of capsaicin.

The current amplitudes were normalized to that induced by 1-μM capsaicin and plotted against the capsaicin concentration (Figure 1B). The curve was drawn with a least-squares fitting routine using the following equation:

$$I\left(\text{C}\right)\;=\;I_{\max}\text{ × }{\text{C}}^{\text{n}}/{(\text{C}}^{\text{n}}\;+{\text{EC}}_{50}^{\text{n}})$$ (1)

where *I* denotes the relative current at concentration C, *I*_max_ the maximal current, EC_50_ the half-maximal concentration and n the Hill coefficient.

**FIGURE 2:**
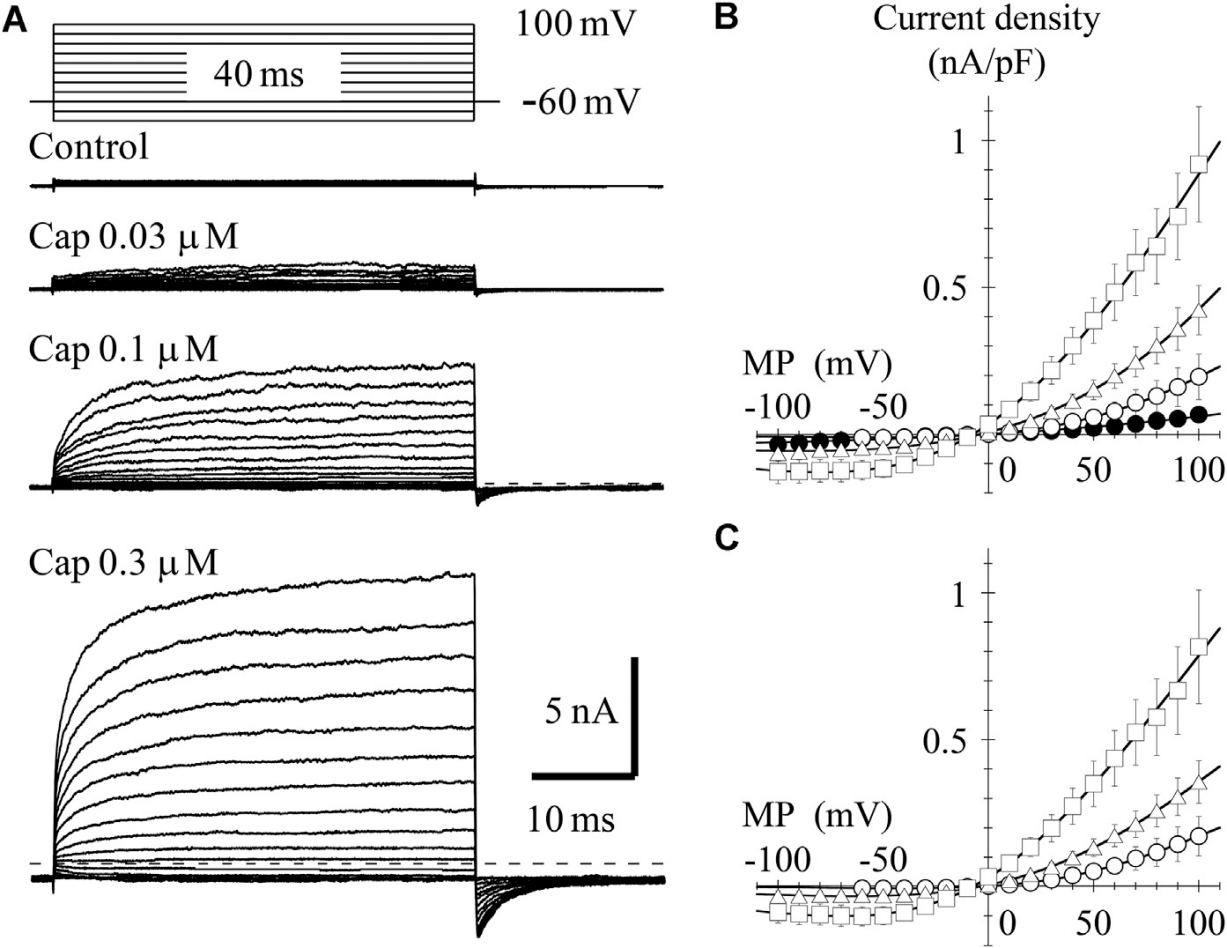
Current–voltage (I–V) relationships for capsaicin-evoked currents. **(A)** Representative whole-cell currents activated by 40-ms voltage steps between −60 or −100 and +100 mV in 10-mV increments from a V_H_ of −60 mV in the presence and absence of capsaicin. The dashed line indicates the zero-current level. **(B)** The averaged current densities of 3-8 cells were plotted against test potentials. Control (closed circle), 0.03- (open circle), 0.1- (open triangle), and 0.3-μM capsaicin (open square). **(C)** Subtracted I-V relationships. Current densities in the presence of capsaicin were subtracted by that in the absence of capsaicin. Curves were fitted using Eq. 2. The G_max_ (1/Ω pF), E_rev_ (mV), E_0.5_ (mV), and *k*_*a*_ (mV) were −0.002, 1.6, 59.3, and 40.7 for 0.03-μM capsaicin, −0.005, −2.5, 44.0, and 54.3 for 0.1-μM capsaicin, and −0.008, −7.0, 1.6, and 50.5 for 0.3-μM capsaicin, respectively.

To determine the current-voltage (I-V) relationship, currents were evoked by 40-ms step pulses from −100 to 100 mV in 10-mV increments every 10 s except for capsaicin 0.03 μM (from −60 to 100 mV). The peak I-V relationship in the presence of 0.03-μM capsaicin shows outward rectification. The averaged current densities (= current amplitude/cell capacitance) are plotted against the test potentials in Figure 2B. Current densities induced by 0.03-, 0.1- and 0.3-μM capsaicin, subtracting current densities in the absence of capsaicin from that in the presence of capsaicin, were plotted against the test potentials as shown in Figure 2C. Solid curves were drawn based on the following equation.

$$I\left(\text{x}\right){=\text{ G}}_{\text{max}}\text{ × }\left(\text{x }-{\text{ E}}_{\text{rev}}\right)/\left\{1\;+\text{ exp }\left[\left({\text{E}}_{\text{0}\text{.}5}\;-\text{ x}\right)/k_{a}\right]\right\}$$ (2)

where G_max_ denotes maximum conductance and E_rev_, E_0.5_, and *k*_*a*_ are the reversal potential, half activation voltage, and slope factor, respectively. The rectification became clear without changing the reversal potential at higher capsaicin concentrations. We were unable to record the outwardly rectifying I-V relationship in TRPV1-expressing HEK293 cells in the absence of capsaicin (control) (Figure 2), nor in the vector-control HEK293 cells in the presence of capsaicin (data not shown).

In the presence of 0.3-μM capsaicin, the time-independent component seems to decay following at potentials around −20 and −10 mV (Figure 2A). Ahern and Premkumar (2002) (see their Figures 1A and 6A) also recorded the decay of current at −40 mV. This decay component is not capacitive current, because it was not recorded in control current in the absence of capsaicin (Figure 2A control). One possible explanation is gating charge. The TRP channels do not have S4 helix like the voltage-gated ion channels, but the charge movement could be recorded by depolarizing pulse around −20 mV during sustained TRPV1 activation. We need additional experiments to prove the hypothesis.

### Activation Kinetics of Transient Receptor Potential Vanilloid-1 Channel in the Presence of Capsaicin

**FIGURE 3:**
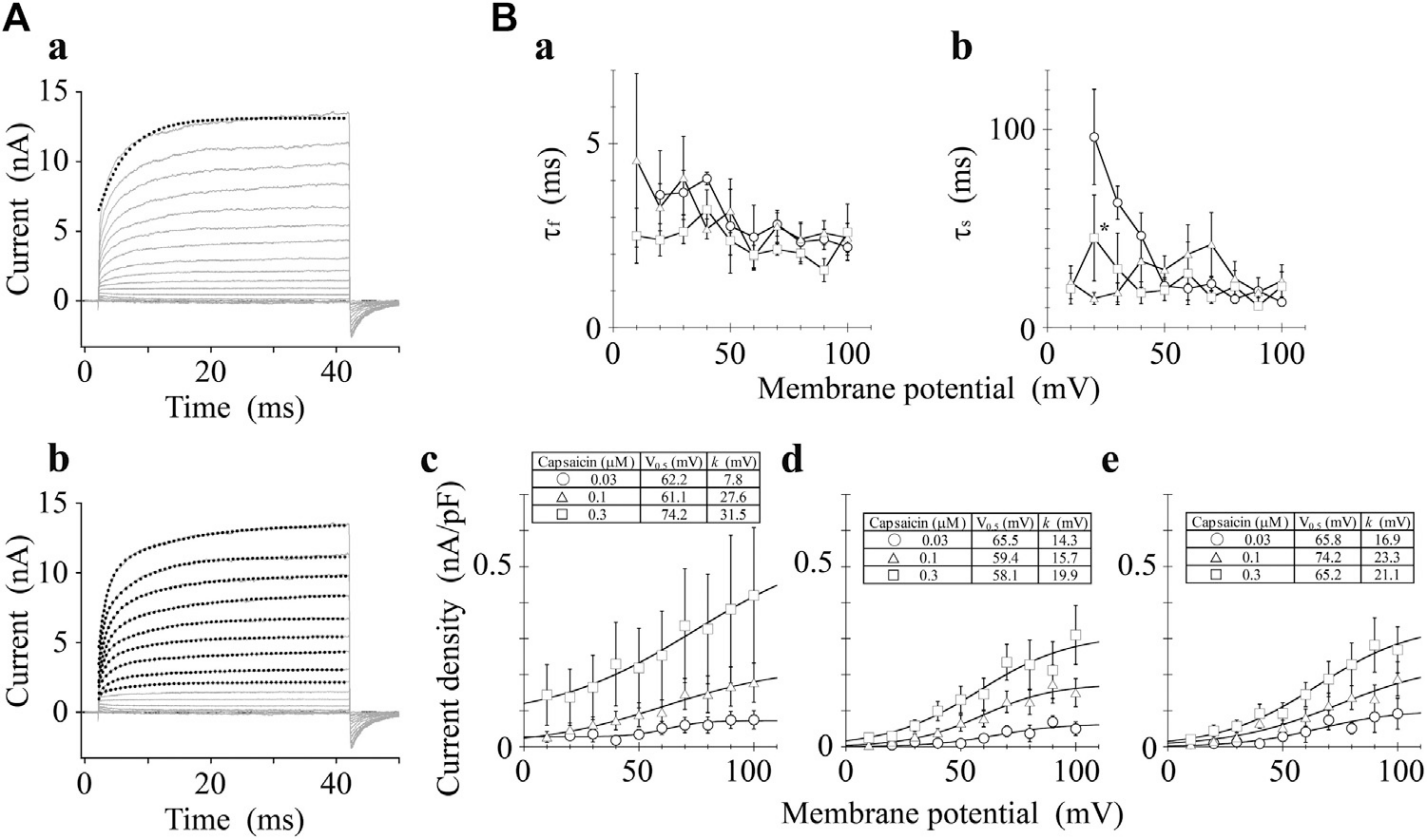
Curve fit of TRPV1 current evoked by both capsaicin and step depolarization. The activation phase evoked by 40-ms step pulse to 100 mV was fitted by a single-exponential function (broken line curve **(A)** (*a*)) and by two exponential functions (broken line curve **(A)** (*b*)). The double-exponential function fits better than the single exponential function. Capsaicin concentration was 0.3 μM. **(B)** Voltage- and concentration-dependence of activation parameters. The five parameters were plotted against the test potentials: (*a*) fast time constant (*τ*_f_), (*b*) slow time constant (*τ*_s_), (*c*) current density of time-independent component, (*d*) current density of fast component, and (*e*) current density of slow component. Curves were drawn by Eq. 2 with a basal component. Data are expressed as the mean ± S.E. of 3, 8 and 5 cells for 0.03- (open circle), 0.1- (open triangle), and 0.3-μM capsaicin (open square), respectively. *p < 0.05 between 0.1- and 0.3-μM capsaicin at 20 mV. The statistical analysis was made using two-way repeated-measures analysis of variance (ANOVA) followed by Tukey’s multiple comparisons tests.

We analyzed the activation time constants of the outward TRPV1 channel currents evoked by step depolarizations. The activation phase of the current at 100 mV fit well to two exponential functions with a time-independent component compared to a single exponential function with a time-independent component (Figure 3A). Current onset occurred more quickly as the test depolarization increased. The time constants at different capsaicin concentrations were not significantly different at 40 and 100 mV.

The current densities of the time-independent, fast, and slow components increased in accordance with the test depolarization (Figures 3Bc–e). The ratios of the three components at a test potential of 40 and 100 mV were summarized in Table 1. In agreement with the growing voltage-steps from 40 to 100 mV, the percentage of the time-independent components decreased by about 10%. To compensate for this reduction, the ratio of the fast and slow components increased. The ratio of the fast component mainly increased during the application of 0.3-μM capsaicin, while the slow component increased during the application of 0.03- or 0.1-μM capsaicin. In other words, capsaicin at higher concentrations accelerates the depolarization-evoked outward currents due to the increase of the ratio for the faster transition component.

**TABLE 1:** Capsaicin concentration-dependent changes (%) of the three components.

	Capsaicin (μM)	n	% at 40 mV			% at 100 mV			Delta
Time- independent	0.03	3	51.6	±	6.9	40.7	±	5.1	−10.9
	0.1	8	39.5	±	3.5	30.0	±	3.4	−9.5
	0.3	5	43.8	±	5.2	34.0	±	5.0	−9.8
τ-fast	0.03	3	23.0	±	7.5	21.6	±	7.2	-1.4
	0.1	8	25.8	±	3.7	28.1	±	2.8	2.3
	0.3	5	27.6	±	3.3	35.0	±	3.8	7.4
τ-slow	0.03	3	25.4	±	3.9	37.7	±	7.2	12.3
	0.1	8	34.8	±	5.0	42.0	±	3.0	7.2
	0.3	5	28.6	±	4.7	31.0	±	5.6	2.4

### Current-Voltage Relationship of Transient Receptor Potential Vanilloid-1 Channel Activated by Protons

**FIGURE 4:**
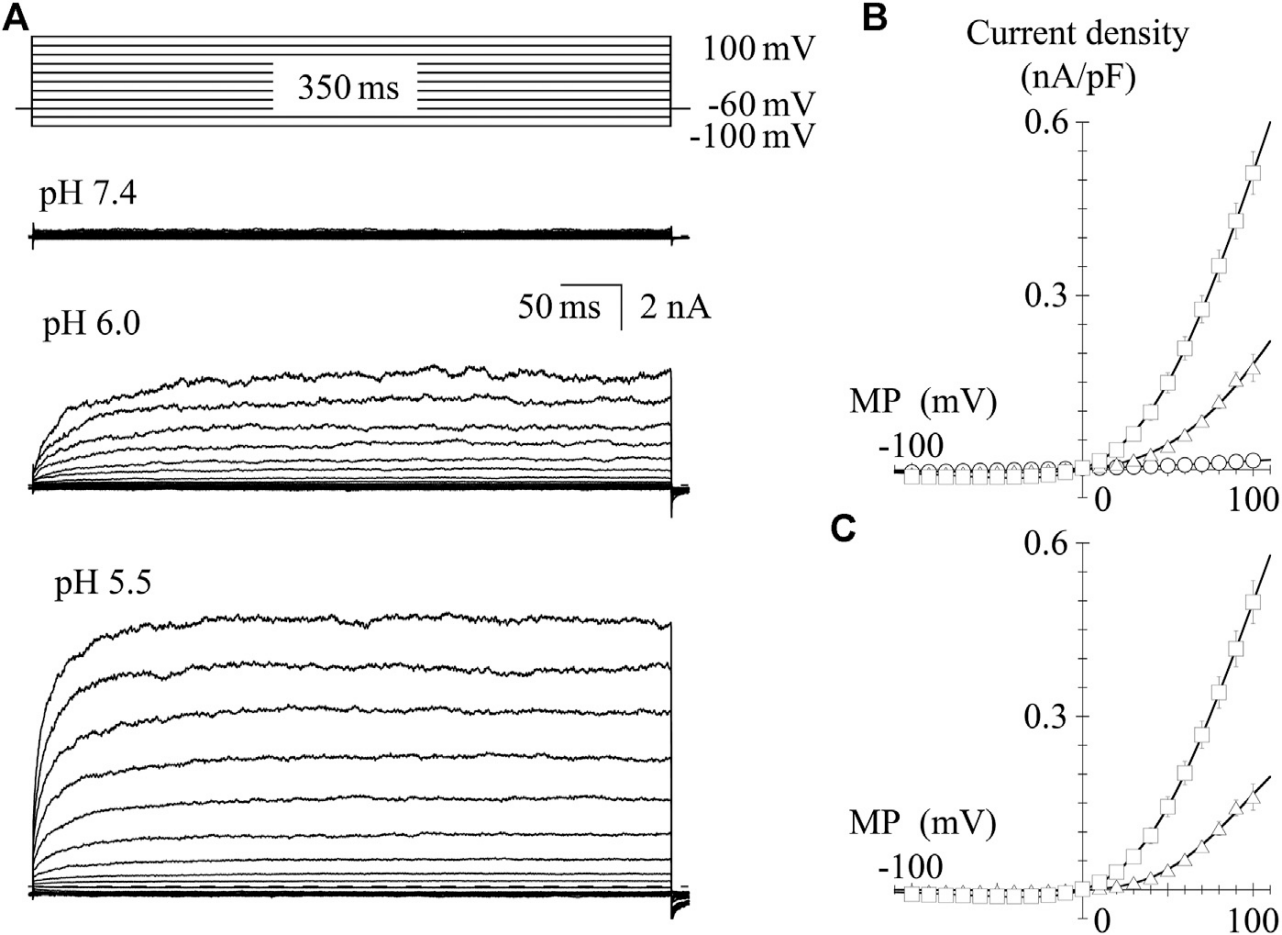
I-V relationships for proton-evoked currents. **(A)** Representative whole-cell currents activated by 350-ms voltage steps to between −100 and +100 mV in 10-mV increments from a V_H_ of −60 mV in the pH 7.4, 6.0, and 5.5 external solutions. The dashed line indicates the zero-current level. **(B)** The averaged current densities were plotted against the test potentials. Data points represent the mean ± S.E. of 10–11 cells. pH 7.4 (open circle), pH 6.0 (open triangle), and pH 5.5 (open square). **(C)** Subtracted I-V relationships. Current densities in the low pH external solutions were subtracted by that in the normal Tyrode solution with a pH of 7.4. Curves were fitted using Eq. 2. The values of G_max_, E_rev_, E_0.5_, and *k*_*a*_ were −0.002, 5.5, 65.7, and 26.3 for pH 6.0 and −0.006, 0.3, 55.1, and 32.5 for pH 5.5, respectively.

TRPV1 channel current is induced by protons; thus, we recorded the I-V relationship in GFP-positive HEK293 cells under the low pH conditions. In the pH 6.0 external solution, protons induced a small inward current at a V_H_ of −60 mV. Protons induced larger inward currents in the pH 5.5 external solution compared to those in the pH 6.0 external solution (see the baseline levels before step pulses, Figures 4A,B). To determine the I-V relationship, currents were evoked using 350-ms step pulses from −100 to 100 mV with 10-mV increments every 10 s. Because of the slower activation under low pH conditions, longer step pulses were necessary to obtain steady-state current levels compared to when effects of capsaicin were evaluated. Peak I-V relationships in the external solutions at pH 6.0 and 5.5 showed outward rectification. The average current densities were plotted against the test potentials (Figure 4B). Current densities induced by protons, subtracting the current densities at pH 7.4 from the densities at pH 6.0 or 5.5, were plotted against the test potentials (Figure 4C). The solid curves were drawn using Eq. 2. The currents reversed around 0 mV and the I-V relationships showed outward rectification like those shown in Figure 2.

### Activation Kinetics of Transient Receptor Potential Vanilloid-1 Channel Under Low pH Condition

**FIGURE 5:**
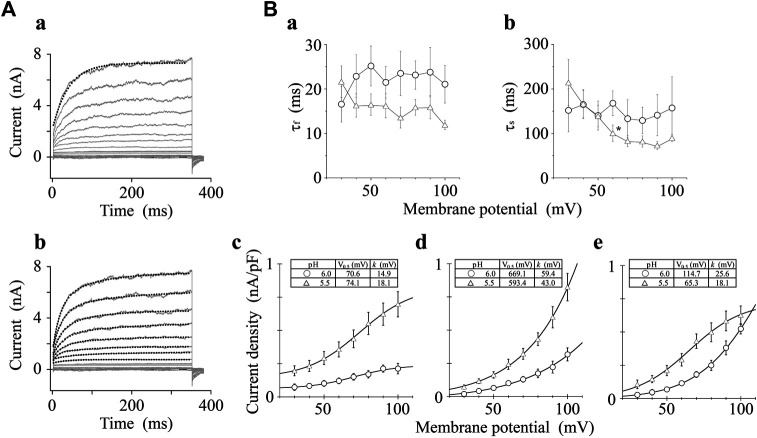
Curve fit of TRPV1 current evoked by both protons and step depolarization. The activation phase evoked by 350-ms step pulse to 100 mV was fit to a single-exponential function (broken line curve **(A)** (*a*)) and to a sum of two exponential functions (broken line curve **(A)** (*b*)). The double-exponential function showed a better fit than the single exponential function. pH was 5.5. **(B)** Voltage- and pH-dependence of activation parameters. The five parameters were plotted against the test potentials and curves were fitted as shown in Figure 3B. Data are expressed as the mean ± S.E. of 10 and 11 cells for pH 6.0 (open circle) and 5.5 (open triangle), respectively. *p < 0.05 between pH 6.0 and 5.5 at 60 mV. The statistical analysis was made using Student’s *t*-test.

We analyzed the activation time constants of the outward TRPV1 channel currents evoked by step depolarizations under low pH conditions in the same manner as shown in Figure 3. The activation phase of the current at 100 mV fit better to two exponential functions with a time-independent component than to a single exponential function with a time-independent component (Figure 5A). The current densities of the time-independent, fast, and slow components increased in accordance with the test depolarization (Figures 5Bc–e). The ratios of the three components at a test potential of 50 and 100 mV were summarized in Table 2. The percentage of the time-independent components was decreased by 20 and 12% with the growing voltage-steps from 50 to 100 mV at pH 6.0 and 5.5, respectively. To compensate for this reduction, the ratio of the fast component increased at pH 5.5, and the ratio of the slow component increased at pH 6.0.

**TABLE 2:** pH-dependent changes (%) of the three components.

	pH	n	% at 50 mV			% at 100 mV			Delta
Time- independent	6.0	10	41.7	±	3.8	21.6	±	2.9	−20.1
	5.5	11	43.0	±	2.0	30.9	±	1.9	−12.1
τ-fast	6.0	10	29.6	±	3.6	30.8	±	3.4	1.2
	5.5	11	27.1	±	2.3	38.5	±	1.9	11.4
τ-slow	6.0	10	28.7	±	2.4	47.6	±	3.3	18.9
	5.5	11	29.9	±	2.3	30.6	±	1.9	0.7

## Discussion

Several groups have recently analyzed voltage-dependent activation of the TRPV1 channel. However, this is the first systematic comparison of the voltage-dependent activation kinetics of TRPV1 channel in the presence of TRPV1 channel activators, capsaicin or protons, using the patch-clamp method. In this study, we found that an outward current in the presence of capsaicin was evoked faster than that in the presence of protons, although both outward phases consisted of a sum of two exponential functions.

### Current-Voltage Relationships

We used a simple index of rectification, specifically the ratio of capsaicin-induced and low pH-induced current at −60 mV and +60 mV (*I*_*+60*_/*I*_*−60*__*mV*_). The rectification ratios were 8.2 ± 2.1, 10.3 ± 1.4, 5.2 ± 1.8, 24.0 ± 6.7, and 23.4 ± 3.9 for 0.03- (*n* = 3), 0.1- (*n* = 8), 0.3-μM capsaicin (*n* = 5), pH 6.0 (*n* = 10) and 5.5 (*n* = 11), respectively, indicating that outward rectification is steeper for pH 6.0 and 5.5 than for capsaicin. However, the rectification indices among the three different capsaicin concentrations and between the low pH solutions were not significantly different.

Current densities at −60 mV were −8 ± 4 pA/pF for 0.03-μM capsaicin (*n* = 3), −33 ± 10 pA/pF for 0.1-μM capsaicin (*n* = 8), −104 ± 32 pA/pF for 0.3-μM capsaicin (*n* = 5), 4 ± 1 pA/pF at pH 6.0 (*n* = 10), and 11 ± 2 pA/pF at pH 5.5 (*n* = 11). The current induced by capsaicin at 0.1 μM was significantly larger than those induced under low pH (*p* < 0.05), suggesting that capsaicin more effectively depolarizes neurons compared to the depolarization by protons. Further, capsaicin can permeate barriers such as the skin and mucosa because of its lipophilicity. Taken together, capsaicin induces pain sensation more potently than protons. The binding sites of protons are the extracellular amino acid residues, E600 and E648, which locate in the linker between the transmembrane domains S5 and S6, the pore-region (Jordt et al., 2000). It is likely that these amino acid residues in the outer mouth of the pore produce the proton-induced smaller inward current at −60 mV and the steeper outward rectification than those induced by capsaicin.

The recombinant rat TRPV1 channel can be activated strongly by depolarizing voltage steps (>50 mV) in the absence of an agonist (Vlachová et al., 2003; Matta and Ahern, 2007). As shown in Figures 2, 4, depolarizing the step pulse to 100 mV from a V_H_ of −60 mV induced no outwardly rectifying currents in our recombinant expression system in the absence of an agonist at room temperature. This discrepancy is partly related to the difference in phosphorylation level in the TRPV1 channel, as PKA (Bhave et al., 2002) and PKC (Premkumar and Ahern, 2000; Bhave et al., 2002) induces TRPV1 channel activity.

### Kinetics of Outward Currents

In the presence of capsaicin or protons, outward currents of the TRPV1 channel were evoked by depolarizing step pulses over approximately 30 mV. Their activation phases were fitted to a sum of two exponential functions. The fast and slow time constants at 100 mV in the presence of capsaicin were respectively 5- and 8-fold lower than those obtained under low pH conditions; however, those among the three different capsaicin concentrations used were not statistically different at > 30 mV (Figures 3Ba,b), and those between the two different pH levels were not statistically different except at 60 mV (Figures 5Ba,b). The tail currents, induced by repolarization to −60 mV, were fitted by a sum of two exponential functions. The fast and slow time constants of the deactivation phases, induced by repolarization from 100 mV to −60 mV, were 0.54 ± 0.26 and 2.92 ± 0.60 (*n* = 3) for 0.03-μM capsaicin, 0.54 ± 0.15 and 3.58 ± 0.39 for 0.1-μM capsaicin (*n* = 8), and 0.78 ± 0.30 ms and 3.48 ± 0.31 ms for 0.3-μM capsaicin (*n* = 5), respectively (data not shown). The fast and slow deactivation time constants among the three different capsaicin concentrations were not statistically different (*p* > 0.05). Further, the two time constants obtained from repolarization from different voltages to −60 mV were voltage-independent. These results suggest that there are at least two closed states and three open states of the channel including the time-independent open state. The recent excellent work using the electron cryo-microscopy (cryo-EM) reveals that the TRPV1 channel opening by capsaicin is associated with dilation of a hydrophobic constriction at the lower gate (Cao et al., 2013). The cryo-EM provides the information of the static structural conformations. The kinetic states include the transient waypoints between the static conformations, and from conformational changes of voltage sensor to those of the lower gate. Opening rate constants depend on neither capsaicin nor proton concentration. The activation time constant should peak around the half-activation voltage in the classical voltage-gated channels such as the P-type Ca^2+^ channel (Wakamori et al., 1998b), however the time constants did not show clean voltage-dependence (Figures 3B, 5B, a,b). These results agree with high number of slope factors (40.7–54.3 mV for capsaicin in Figure 2C, 26.3–32.5 mV for acidic condition in Figure 4C) and lack of clear voltage sensor in the TRPV1 such as the typical voltage-gated channels. Current amplitudes of the time-independent, fast and slow components increased in accordance with the depolarization (Figures 3B, 5B, c–e). The contribution of the time-independent component to the total current decreased as with increasing membrane depolarization, but that of the slow components increased as with increasing membrane depolarization during the application of capsaicin at low concentration and weakly acidic solution (pH 6.0). High concentration capsaicin and acidic solution (pH 5.5) increased in the ratio of fast components (Tables 1, 2).

The slope factors for the fast and slow components were >14 mV (Figures 3B, 5B), which is three times larger than those for voltage-gated Ca^2+^ channels (around 5 mV) (Wakamori et al., 1998a; 1998b). This weak voltage-dependency is accounted for by the fact that the transmembrane domain S4 of TRPV1 channel does not contain positively charged amino acid residues at regular intervals such as the voltage-gated Na^+^, Ca^2+^, and K^+^ channels (Caterina et al., 1997; Caterina et al., 1999; Tao et al., 2010; Szolcsányi and Sándor, 2012).

Y512 and S513 in the S3 segment, T551 in the S4 segment, and E571 in the S4-S5 linker in the mouse TRPV1 form hydrophobic capsaicin-binding pocket (Yang et al., 2015). On the other hand, proton cannot access the capsaicin-binding pocket (Gavva et al., 2005). E600 and E648 at extracellularly facing S5-S6 linker are binding sites for proton (Jordt et al., 2000). Interestingly, permeant cations, Ca^2+^ and Mg^2+^, also bind to the proton binding sites (E600 and E648), and then sensitize capsaicin receptor. Both the cations at higher concentrations themselves activate the TRPV1 channel (Ahern et al., 2005). Although the capsaicin binding sites and proton binding sites are localized in the different areas of TRPV1, both capsaicin- and proton-driven activations link to the facilitation of voltage-dependent TRPV1 channel activation. Binding of capsaicin and proton induces a conformation changes in the capsaicin-binding pocket and the S5-S6 linker, respectively. These structure changes may allosterically modulate voltage sensor of the TRPV1 channel. Although the clear voltage-sensor has not been identified in TRPV1, the capsaicin-binding pocket may be close to the voltage sensor. Further work should be aimed at defining the voltage sensor like the S4 segment of classical voltage-gated channels.

The time-dependent activation of the TRPV1 channel by step depolarization and the broad voltage-dependent activation suggest that the TRPV1 channel slowly drives the feed-forward cycle of pain sensation. Coincident and sustained spikes induced by the TRPV1 channel facilitate TRPV1 channel activity, which is similar to the N-methyl-d-aspartate receptor during long-term potentiation (Huganir and Nicoll, 2013), but different from other pain-related receptors and channels expressed in nociceptors.

## Data Availability

The original contributions presented in the study are included in the article/Supplementary Material, further inquiries can be directed to the corresponding author.
